# Strategies to Deal With Suicide and Non-suicidal Self-Injury in Borderline Personality Disorder, the Case of DBT

**DOI:** 10.3389/fpsyg.2018.02595

**Published:** 2018-12-17

**Authors:** Paco Prada, Nader Perroud, Eva Rüfenacht, Rosetta Nicastro

**Affiliations:** TRE Unit, Division of Psychiatric Specialties, Department of Mental Health and Psychiatry, Geneva University Hospitals, Geneva, Switzerland

**Keywords:** borderline personality disorder, suicide attempts, dialectical behavioral therapy, self-harm, psychotherapy

## Abstract

One of the most problematic aspects of borderline personality disorder resides in repeated non-suicidal self-injury (NSSI) and suicide attempts. These behaviors constitute the prime therapeutic target and a factor that complicates patient care, namely in terms of therapeutic continuity. It has been demonstrated that Dialectical Behavior Therapy (DBT) is efficient in reducing the symptomatology of this disorder, as well as NSSI and suicide. DBT is a multi-component psychotherapeutic treatment, and the effectiveness of its individual constituents is therefore a relevant question. Studies comparing its various components (individual therapy, group therapy, and standard DBT) have not revealed any marked difference between them, other than a tendency toward improved patient retention rates in the standard version of the treatment. The aim of this study is to review the various components of DBT and their constituent parts, in order to highlight the importance of focusing on self-harm behaviors within the therapy as a whole. Although therapeutic strategies may differ and target directly suicide or NSSI, managing the quality of life, and the persistence of the therapeutic alliance (and of the interpersonal alliance) is equally important in terms of treatment efficacy.

## Introduction

Borderline Personality Disorder (BPD) is a disorder affecting from 1 to 6% of the population (Torgersen et al., 2001; Gross et al., 2002; Lenzenweger et al., 2007). Despite a relatively favorable spontaneous evolution, with 80% of patients no longer meeting the DSM diagnostic criteria after a 10-year period, it nonetheless gives rise to significant suffering and functional limitations (Skodol et al., 2002; Zanarini et al., 2006; Niesten et al., 2016). The associated suicide rate is tragically high and estimated at 8–10% (Oldham, 2006). Over the course of the past two decades, various therapies specifically adapted to this disorder have been developed and include Dialectical Behavior Therapy (DBT), Mentalization-Based Treatment (MBT), Transference Focused Psychotherapy (TFP) and Good Psychiatric Management (GPM) (Linehan et al., 1991; Clarkin et al., 2001; Bateman and Fonagy, 2010; Links et al., 2015). Each treatment relies on a consistent theoretical model and provides psychotherapeutic interventions that target the disorder’s central dimensions, which include a perturbed sense of identity, intense and unstable interpersonal relations, emotional dysregulation, poor impulse control and suicidal, and self-harm behaviors (Lieb et al., 2004; Leichsenring et al., 2011).

## Non-Suicidal Self-Injury and Suicide

Non-suicidal self-injury (NSSI) and suicide are the treatment’s primary targets. They are associated with a high rate of emergency care, hospital treatment and outpatient treatment (Crowell et al., 2009). In the past, self-harm was viewed as suicide equivalent, but clinicians have gradually started drawing purpose-based differences between these behaviors in order to provide specific therapeutic responses (Linehan et al., 1991).

Between 17 and 80% of BPD patients are prone to repeated NSSI (the most frequent being self-injury by cutting or burning), and for the same population, suicide attempts range from 46 to 92% (Zanarini et al., 2008). NSSI has a higher prevalence among adolescents and young adults, and is associated with an array of psychosocial issues and is believed to be a transdiagnostic manifestation arising from an underlying vulnerability inherent to the psychopathology (Crowell et al., 2009; Selby et al., 2015). NSSI differs from suicide in that it is not associated with suicidal inclinations: furthermore, the strategies implemented for behaviors aiming at regulating negative emotions are different from those used to manage behaviors aiming at causing death (Crowell and Kaufman, 2016). Unlike suicide, NSSI has an immediate and short-term impact, and must be carried out repeatedly to produce the desired outcome (Shaffer and Jacobson, 2009). Furthermore, patients who are disposed to NSSI generally resort to other methods when attempting suicide, a fact that suggests that suicide is not a severe manifestation of NSSI, and that NSSI does not amount to failed suicide (Stanley et al., 2001). However, NSSI and suicide are bound by a complex relationship, and it is often difficult to draw a clear distinction between both categories. Therefore, individuals do not always fit into one category or the other. In addition, some patients whose intention to die is moderate use highly lethal means, and inversely individuals who report highly intense suicidal urges use non-lethal means (Crowell and Kaufman, 2016). The presence of NSSI is the strongest predictor of future suicide attempts. Indeed, a study has shown that 70% of adolescents with NSSI report a suicide attempt in the course of their life (Nock et al., 2006). This tragic relationship is supported by further evidence, since 1.8% of patients presenting NSSI commit suicide within 1 year following the incident (Owens et al., 2002), and up to 8.5% commit suicide within a 22-year period (Jenkins et al., 2002). Finally, it appears that NSSI has some protective value, as long as it provides relief from mental suffering during dissociative episodes (as a coping strategy) (Kliem et al., 2010).

Several theoretical elaborations have examined the passage from suicidal ideation to suicide. These “ideation to action theories,” such as the interpersonal theory of suicide (IPTS) by Joiner, and the three-step theory (3ST) by Klonsky, highlight the role of pain, of hopelessness and of perceived interpersonal disconnection in the emergence of suicidal ideation. People who commit suicide differ from those who merely entertain suicidal thoughts by their capability for suicide. In particular, this capability is acquired through traumatic life experiences and NSSI (Klonsky et al., 2018).

Studies reveal that the efficacy of therapeutic interventions is improved when NSSI and suicide are considered globally and that the effect of therapies seems stronger on NSSI (Ougrin et al., 2015).

Dialectical behavior therapy has been shown to have a statistically significant effect on the reduction of personality disorder symptoms (Kliem et al., 2010; Cristea et al., 2017). For NSSI, a number needed to treat of 14 to prevent 1 incident is better than in other medical intervention (Ougrin et al., 2015). Patients receiving psychotherapeutic treatment are less likely to attempt suicide than patients receiving treatment as usual, in particular for those who suffer from BPD (Calati and Courtet, 2016).

## Dialectical Behavioral Therapy and Suicide

Dialectical behavior therapy is a structured cognitive-behavioral treatment initially designed to treat chronically suicidal patients suffering from BPD. It is based on a biosocial theory of emotion dysregulation. Simply put, BPD’s pervasive emotion dysregulation is a consequence of repeated transactions during child development between biological vulnerabilities and an invalidating environment. The term “dialectical” refers to a philosophy defining the nature of reality, and to persuasive dialog and relationship. Dialectics are at the heart of the therapy, from the very conceptualization of emotional dysregulation to the implementation of DBT core strategies. For instance, the balance between acceptance and change is crucial in DBT. Core strategies include change-oriented interventions like contingency management or cognitive restructuring and acceptance-orientated interventions including the practice of mindfulness. These skills are implemented in a constant effort to strike a dialectical balance between accepting the reality as it is, and changing behaviors that need to be changed. The four treatment modes of standard DBT described herein are as follows: individual therapy, group skills training, telephone coaching, and the therapist consultation team. Each plays a role in the therapy’s main functions (Linehan et al., 1991, 2015), which are: (1) Improving the patient’s skills, (2) Extending these skills to the environment, (3) Increasing and maintaining the patient’s motivation to change, (4) Increasing the therapist’s motivation and skills, and (5) Structuring the environment to optimize the implementation of the treatment.

Although treating NSSI is a priority in the hierarchy of therapeutic targets, it is not explicitly mentioned as one of the therapy’s main functions. It is theorized that these behaviors are reduced or eliminated as a result of a range of factors, such as the acquisition of skills in various areas, and the acceptance of life’s difficulties. In DBT, NSSI and suicidal behaviors are viewed as responses to unbearable emotional suffering, and though they may be dysfunctional, they are highly effective emotion regulation strategies. Indeed, they dramatically reduce emotion intensity and immediately relieve from intense suffering. Besides, they can generate social consequences, such as obtaining more presence and help from the environment, or justifying a hospital admission. DBT aims to change dysfunctional patterns in emotion regulation, impulse control, identity and interpersonal relationships to construct a life worth living. Emotionally dysregulated patients are encouraged to change their maladaptive problem-solving strategies by learning skills to tolerate distress with more effective and adaptive means. Four skills modules are proposed: mindfulness, distress tolerance, interpersonal effectiveness and emotion regulation.

Dialectical behavior therapy has been shown to be effective in terms of its therapeutic objectives: reducing NSSI and suicide, reducing therapy-interfering behaviors as well as behaviors interfering with the quality of life (Linehan et al., 1991, 2006; Bohus et al., 2004; Burmeister et al., 2014).

The fact that DBT relies on four treatment modes naturally gives rise to the question of the relative effectiveness of each component. The usefulness of individual and group therapy has been tested separately, without revealing any significant difference in terms of suicide attempts, NSSI occurrence, and the number of hospitalizations or emergency consultations. The superiority of the standard treatment appears to reside in increased patient retention rates – which is a feature of interest given that high dropout rates are one of the greater problems among this group of patients - but also in globally improved mental health (Linehan et al., 2015).

However, identifying the key factors driving the efficacy of DBT remains delicate, in particular as far as NSSI or suicide and change mechanisms are concerned (Calati and Courtet, 2016).

## Individual Therapy

Two important factors should be noted: The quality of the therapeutic alliance in DBT, and compliance with the target hierarchy in individual therapy.

In Dialectical behavior therapy, it is essential to establish a therapeutic alliance that combines warmth and structure (Robins and Koons, 2000). For patients, this is often the first positive experience of therapy. The demeanor, attitude and words of the therapist should be considered as therapeutic in themselves, as they encourage the creation of a relationship stemming from understanding, validation and trust, which in turn increases motivation and promotes therapeutic change. From the therapist’s point of view, considering the BPD in a non-judgemental and empathic way is conducive to collaboration, despite difficult behaviors that can push the therapist to his or her limits. This kind of relationship helps increase patient retention rates (Linehan et al., 2015).

In a study pertaining to therapeutic alliances and introjection comparing DBT and community treatment by experts (Bedics et al., 2012), patients reported major improvements in terms of self-affirmation, self-love, self-protection, as well as fewer instances of self-attack, during the course of treatment and the 1-year follow-up period. The quality of the therapeutic alliance combines with the treatment itself to yield positive results. Patients undergoing DBT who perceived their therapist as self-assured and protective reported fewer cases of NSSI.

## Nssi as Primary Target

Dialectical behavior therapy individual therapists are responsible for the management of suicidal crises. For example, the therapist faced with a suicidal patient can refer to specific DBT strategies including the therapist survival protocol, which defines what to do when the patient is in crisis. The therapist has the responsibility to provide a clear and active response to prevent the patient from committing NSSI or suicide, for example by removing lethal means available to the patient. Mean restriction is a way to reduce the capacity to attempt suicide while teaching the patient to survive the crisis by tolerating acute distress using skills with the support of the therapist.

The structure of individual therapy also helps the therapist to target, directly and overtly, any change in the pattern of ideas and of speech, and any evolution of suicidal behaviors. The individual therapy schedule in DBT is set according to the hierarchy of targets established when the patient and therapist enter into a therapeutic alliance. It relates to specific behaviors that need to be reduced: (1) Life-threatening behaviors, (2) non-collaboration and non-participation behaviors or behaviors that threaten to interfere with the therapy or push the therapist to his/her limits, and (3) behaviors that interfere with the quality of life. Any recent NSSI is thereby systematically addressed as a priority by conducting a behavioral chain analysis. The aim of a chain analysis is to describe the chain of components leading to a specific behavior and to establish a plan for problem solving. It helps to understand what prompts and what maintains the behavior and to figure out what problems must be solved and which skills are missing to stop the behavior. The chain analysis provides information on what motivates NSSI or suicide and allows therapist and patient to figure out how to prevent these behaviors. Motivations for suicide are frequently related to acute emotional distress and hopelessness (internal motivations) or to a need to communicate and influence others (external motivations); internal motivations appear to be more strongly correlated to a desire to die and to a higher level of intent and preparation (Klonsky et al., 2016). The individual therapist has to focus on the patient’s specific motivations for NSSI or suicide and reasons for living, and help him/her apply the needed skills to build a life worth living and reduce internal and external motivations for suicide.

## Skills Training Groups

The goal of skills training is to teach the patient the general skills to help him/her solve problems in living. Skills training is probably a key element of DBT as it directly addresses behavioral, cognitive, emotional, and interpersonal dysfunctional patterns which lead to NSSI and suicide. There are two acceptance skills modules (mindfulness and distress tolerance) and two change skills modules (emotion regulation and interpersonal effectiveness) that are balanced to address one of the major dialectics for the patient: to accept himself/herself and reality as it is, and to change what needs to be changed. Mindfulness skills teach to focus attention on the present moment and to observe reality as it is. It addresses patient’s dysregulation of the sense of self and reduces the feeling of being disconnected from others. Distress tolerance skills address the ability to tolerate and survive crisis situations and to accept painful events or emotions, in order to reduce suffering. These skills are essential to avoid NSSI which are often related to overwhelming suffering. Interpersonal effectiveness skills help maintain and improve relationships, deal with conflict situations and obtain what the person needs while maintaining self-respect. These skills are necessary to solve interpersonal conflicts without having to rely on extreme and dysfunctional behaviors such as threats or verbal aggression. The goals of emotion regulation skills are to improve emotion observation and understanding and to reduce emotional vulnerability. The skills acquisition allows patients to achieve better emotional and behavioral control, which translates primarily into reduced NSSI and suicide (Neacsiu et al., 2010). Moreover, two studies showed that DBT with skills training is more efficient in reducing NSSI than DBT without skills training (Linehan et al., 2015; Euler et al., 2018).

Furthermore, by sharing personal experiences with others (during groups), bonds can be created, with the probable outcome of increasing the feeling of interconnectedness, in a context of reinforcement and non-judgement.

## Telephone Coaching

In Dialectical behavior therapy, phone calls are encouraged for many reasons (Linehan, 1993). Firstly, BPD patients often have difficulties in asking for help and will rely on extreme behaviors, such as NSSI and suicide, to ask for help or to increase presence and support from the environment. Secondly, patients need help to implement and generalize skills acquired during group sessions into everyday life. The patient is encouraged to call before engaging in NSSI so that he or she may receive coaching and be reminded to use his or her skills. Telephone coaching is an essential prevention means as it focuses on NSSI and suicidal risk assessment and the use of alternative solutions to solve the problem. Phone calls are also an effective way of healing the therapeutic alliance and improving patient satisfaction and reducing the risk of therapy dropout (Chalker et al., 2015). It seems obvious that the availability of the therapist outside scheduled individual sessions helps develop a sense of security and connectedness for the patient, and probably for the therapist too. A study (Coyle et al., 2018) shows that visits to crisis centers were linked to suicide risks. DBT, with its telephone coaching mode and skills training, namely in terms of distress tolerance strategies, helps reduce dependence on these services, indirectly lowering the risk of suicide (Stanley et al., 2001).

## Consultation Team

Dialectical behavior therapy assumes that an effective therapy should pay as much attention to the therapist as it does to the patient; that is why the consultation team is an integral component of DBT. Each DBT therapist must be part of a consultation team and attend weekly meetings. The consultation team is responsible for helping the therapist to stay motivated and within the DBT therapeutic frame, applying DBT strategies and techniques to maintain an effective therapeutic relation with the patients. The consultation team encourages adherence to DBT philosophy and allows therapists to consolidate their skills and receive support, strengthening the implementation of DBT in the care they provide, and within the treatment team. Therapists who treat BPD patients need this supervision because they are often reinforced by the patient in engaging in ineffective behaviors, while being punished when engaging in effective behaviors (Linehan, 1993). For example, the patient becomes very friendly when the therapist gives up the hierarchy of targets allowing him/her to avoid talking about a recent NSSI. Furthermore, progress is slow and the therapist can become vulnerable to criticism or discouragement. The consultation team refers to dialectics, uses encouragements, avoids judgment or team divide and searches for alternative solutions when needed. Regarding NSSI and suicide behaviors, therapists are often confronted with intense emotions and such extreme behaviors can generate a high level of stress. The consultation team plays an important role in helping therapists to observe their limits and avoid a burnout. A recent study on clinicians’ experiences of the consultation team shows that the meetings help maintain the therapists’ motivation (Walsh et al., 2018).

## Discussion and Conclusion

Despite mounting evidence of the positive impact of DBT in the treatment of BPD, the relative effects of specific therapeutic interventions on suicidal behaviors remain difficult to assess. It would appear that some components of DBT, such as skills training groups and individual therapy, have the same positive impact on NSSI (Linehan et al., 2015).

Suicide and NSSI are obviously targets of the therapy and elements that interfere with its prognosis and positive outcome.

Elements that have an impact on reducing NSSI and suicide among BPD patients are numerous and exist at different levels of DBT, in its range of targets, modes, strategies or types of therapeutic intervention, and in the consistency of the theoretical model and therapeutic structure. This is in keeping with various therapeutic models that have shown comparable efficacy, such as MBT, TFP and GPM, and that consider NSSI and suicide as priorities in their treatment structure, while maintaining the fundamental objective of improving quality of life (Keuroghlian et al., 2016).

Nevertheless, we still need to better understand which and how specific components of DBT reduce suicidal behaviors and for which patients. Further researches are clearly needed in this field.

## Perspectives and New Elements

Clinicians caring for patients exhibiting self-destructive behaviors are keenly interested in the use of new technologies. The advantage of such technologies is their immediate availability to patients. Smartphone applications have therefore been developed, either for the benefit of patients (Rizvi et al., 2011; Prada et al., 2017), or to help clinicians treating suicidal patients (Harned et al., 2017).

## Author Contributions

PP, NP, ER, and RN participated in equally in designing the study, in writing the different sections of the paper, and in giving their final approval for the paper to be submitted.

## Conflict of Interest Statement

The authors declare that the research was conducted in the absence of any commercial or financial relationships that could be construed as a potential conflict of interest.
